# Integrative analysis identifies oxidative stress biomarkers in non-alcoholic fatty liver disease via machine learning and weighted gene co-expression network analysis

**DOI:** 10.3389/fimmu.2024.1335112

**Published:** 2024-02-27

**Authors:** Haining Wang, Wei Cheng, Ping Hu, Tao Ling, Chao Hu, Yongzhen Chen, Yanan Zheng, Junqi Wang, Ting Zhao, Qiang You

**Affiliations:** ^1^ Medical Center for Digestive Diseases, Department of Geriatrics, the Second Affiliated Hospital of Nanjing Medical University, Nanjing, China; ^2^ Department of Orthopedics, Tianjin Medical University General Hospital, Tianjin, China; ^3^ Department of Medical Oncology, Shenzhen Traditional Chinese Medicine Hospital, Shenzhen, China; ^4^ Department of Medical Oncology, Fudan University Shanghai Cancer Center, Shanghai, China

**Keywords:** non-alcoholic fatty liver disease, bioinformatic analysis, machine learning, WGCNA, CDKN1B, NDUFA4, TFAM

## Abstract

**Background:**

Non-alcoholic fatty liver disease (NAFLD) is the most common chronic liver disease globally, with the potential to progress to non-alcoholic steatohepatitis (NASH), cirrhosis, and even hepatocellular carcinoma. Given the absence of effective treatments to halt its progression, novel molecular approaches to the NAFLD diagnosis and treatment are of paramount importance.

**Methods:**

Firstly, we downloaded oxidative stress-related genes from the GeneCards database and retrieved NAFLD-related datasets from the GEO database. Using the Limma R package and WGCNA, we identified differentially expressed genes closely associated with NAFLD. In our study, we identified 31 intersection genes by analyzing the intersection among oxidative stress-related genes, NAFLD-related genes, and genes closely associated with NAFLD as identified through Weighted Gene Co-expression Network Analysis (WGCNA). In a study of 31 intersection genes between NAFLD and Oxidative Stress (OS), we identified three hub genes using three machine learning algorithms: Least Absolute Shrinkage and Selection Operator (LASSO) regression, Support Vector Machine - Recursive Feature Elimination (SVM-RFE), and RandomForest. Subsequently, a nomogram was utilized to predict the incidence of NAFLD. The CIBERSORT algorithm was employed for immune infiltration analysis, single sample Gene Set Enrichment Analysis (ssGSEA) for functional enrichment analysis, and Protein-Protein Interaction (PPI) networks to explore the relationships between the three hub genes and other intersecting genes of NAFLD and OS. The distribution of these three hub genes across six cell clusters was determined using single-cell RNA sequencing. Finally, utilizing relevant data from the Attie Lab Diabetes Database, and liver tissues from NASH mouse model, Western Blot (WB) and Reverse Transcription Quantitative Polymerase Chain Reaction (RT-qPCR) assays were conducted, this further validated the significant roles of CDKN1B and TFAM in NAFLD.

**Results:**

In the course of this research, we identified 31 genes with a strong association with oxidative stress in NAFLD. Subsequent machine learning analysis and external validation pinpointed two genes: CDKN1B and TFAM, as demonstrating the closest correlation to oxidative stress in NAFLD.

**Conclusion:**

This investigation found two hub genes that hold potential as novel targets for the diagnosis and treatment of NAFLD, thereby offering innovative perspectives for its clinical management.

## Introduction

Approximately 25% of the global population is afflicted with Non-alcoholic fatty liver disease (NAFLD), although the prevalence varies due to regional disparities. The Middle East (32%) and South America (30%) exhibit the highest rates, while the prevalence is 24% in North America and Europe, 27% in Asia, and the lowest in Africa at 13% (1). The American Association for the Study of Liver Diseases (AASLD) has defined NAFLD in its practice guidelines as: (a) the presence of hepatic steatosis, either by imaging or histology, and (b) no causes for secondary hepatic fat accumulation, such as significant alcohol consumption, use of steatogenic medication, or hereditary disorders (2).

NAFLD can be further subdivided into Non-alcoholic fatty liver (NAFL) and Non-alcoholic steatohepatitis (NASH) (3). NAFL is defined by hepatic steatosis without evidence of hepatocellular injury in the form of hepatocyte ballooning. Conversely, NASH is characterized by hepatic steatosis and inflammation with hepatocyte injury, with or without fibrosis (4). NAFL can transform into NASH, which is characterized by hepatocellular ballooning and lobular inflammation as well as steatosis. Perisinusoidal fibrosis is typically not considered a prerequisite for diagnosing NASH (5). NAFLD may evolve into cirrhosis and hepatocellular carcinoma (HCC) (6), with HCC representing the fourth leading cause of cancer-related deaths worldwide (7). In the United States, NASH is the second most common indication for liver transplantation (8). Among U.S. HCC patients requiring liver transplantation, those with NAFLD represent the fastest-growing group (9), highlighting the substantial disease burden posed by NAFLD.

Oxidative stress (OS) means an imbalance between oxidative and antioxidative processes within an organism. Under these conditions, the quantity of Reactive Oxygen Species (ROS) and Reactive Nitrogen Species (RNS) produced by the organism surpasses its antioxidative capabilities, thereby inducing oxidative damage. ROS and RNS are small molecules with robust oxidative characteristics, encompassing both free radicals and non-free radicals, such as superoxide anions, hydroxyl free radicals, hydrogen peroxide, and nitric oxide. When tissues, cells, and biological macromolecules are exposed to these excessive oxidants over an extended period, a series of biochemical reactions are triggered, causing oxidative damage and consequently, impairing normal cellular functions. Prolonged oxidative stress is regarded as a pivotal factor in instigating various diseases such as cardiovascular diseases (10), cancer (11), neurodegenerative diseases (12), diabetes (13), and aging (14). To prevent oxidative damage, an antioxidative system exists within the organism, consisting of antioxidative enzymes (such as superoxide dismutase and catalase) and non-enzymatic antioxidants (such as vitamin C, vitamin E, and glutathione). This system can neutralize ROS and RNS, shielding cells from their detrimental effects.

In animal experiments, we found that carbon tetrachloride can lead to hepatic fat accumulation and damage. After reviewing the literature, we learned from several studies by Slater et al. that free radicals play a key role in causing liver damage (15). This implies that free radicals play a pathogenic role in initiating liver diseases, while antioxidants have therapeutic effects on free radical-mediated NAFLD (16). Furthermore, epidemiological, clinical, and experimental research targeting the liver reveals that NAFLD is closely associated with alterations in redox status and subsequent increased metabolic risk (17). According to the “second hit” and “multiple hit” theories, oxidative stress appears to be one of the most critical mechanisms causing NAFLD liver injury and plays a vital role in the progression from NAFL to NASH (18). Studies have demonstrated that the liver is a principal organ attacked by ROS (19), where an increase in ROS can induce lipid peroxidation by activating Hepatic Stellate Cells (HSC), thereby resulting in inflammation and fibrosis formation. Moreover, ROS can inhibit hepatic VLDL secretion, inducing hepatic fat accumulation, and also promote hepatic insulin resistance and necrotizing inflammation, activating several cell pathways leading to hepatocyte apoptosis (20). Several interrelated pro-oxidative factors, along with mitochondrial dysfunction, might also contribute to the occurrence of OS. Targeted research on OS represents a promising direction in treating NASH. Inspired by these pioneering studies, we decided to explore the relationship between NAFLD and OS through bioinformatics analysis, hoping to offer new insights and guidance for the clinical diagnosis and treatment of NAFLD.

In this research, based on the results of the Limma package and Weighted Gene Co-expression Network Analysis (WGCNA), we identified 31 genes related to NAFLD and OS. Furthermore, we employed three machine learning algorithms—Least Absolute Shrinkage and Selection Operator (LASSO), Support Vector Machine-Recursive Feature Elimination (SVM-RFE), and RandomForest to examine these genes. The results suggested that CDKN1B, NDUFA4, and TFAM are intimately related to oxidative stress in NAFLD, providing new insights for the diagnosis and treatment of NAFLD.

## Materials and methods

### Data collection and processing

**Figure 1:**
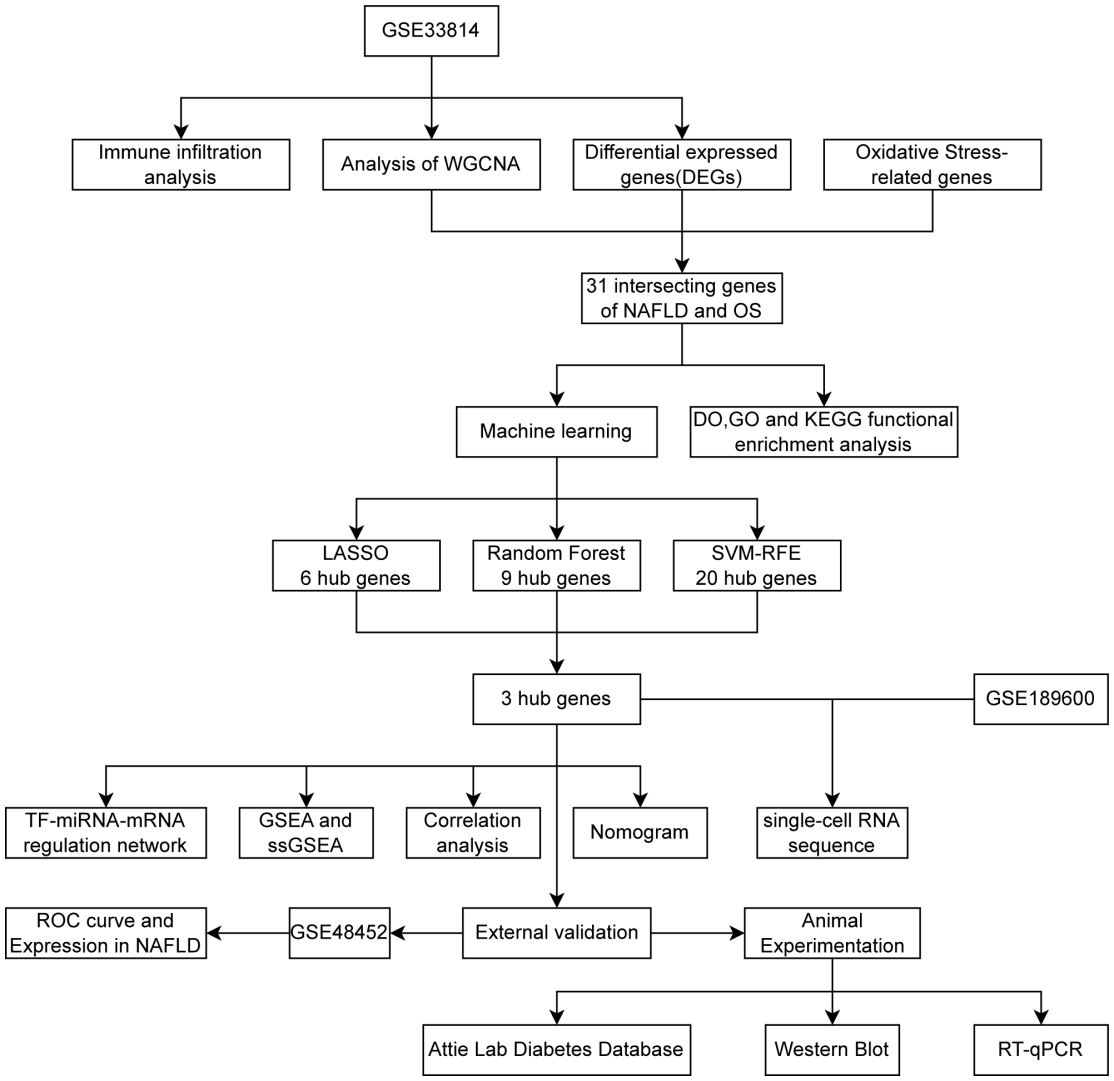
Flowchart 3 hub genes (CDKN1B,NDUFA4,TFAM) identified by three machine learning algorithms.

**Figure 1** was created to show the flowchart of our data analysis process. The datasets GSE33814 (GPL570) and GSE48452 (GPL11532) were retrieved and downloaded from the Gene Expression Omnibus (GEO) database (https://www.ncbi.nlm.nih.gov/geo/). GSE33814 consists of 44 samples: 13 control, 19 NAFL, and 12 NASH. GSE48452 consists of 46 samples: 14 control, 14 NAFL, and 18 NASH, serving as a dataset for validation. It is imperative to note that many studies on NAFLD that undergo bioinformatic analysis selectively utilize samples from the more severe NAFLD stage, NASH, for analysis. For the sake of academic rigor in this study, NAFL samples have been included in addition to NASH samples. The “Limma” package (21) was utilized for normalizing sample data, conducting conversion between probe ID and gene symbols through coding, eliminating probes without gene symbols, and calculating the average expression value under the same symbol.

Genes related to OS were retrieved and downloaded from the Genecard database (https://www.genecards.org/), using a relevance score greater than 7 as a selection criterion (22), resulting in the extraction of 1065 genes associated with oxidative stress.

### Implementation of WGCNA and identification of key module genes

WGCNA is a robust systems biology method, designed for identifying coexpressed gene modules and exploring associations between gene networks and notable phenotypes, along with deciphering key genes within the networks. WGCNA enables the discovery of highly correlated gene clusters, which typically share common functionalities within biological processes. Significantly, WGCNA constructs a weighted network, indicating that connections within the network not only represent a binary existence but also mirror the correlation strength among genes, an essential feature to accurately represent intricate relationships between genes.

In our research, WGCNA, implemented through the R package “WGCNA” (23), was utilized to identify modules with the highest relevance to NAFLD. WGCNA encompasses five primary steps: gene clustering, assessing whether the soft-thresholding power approximates a scale-free network, merging similar modules (with the cut height for merging modules set at 0.25 and MEDissThres = 0.25, implying the merging of modules with a similarity greater than 0.75), associating modules with traits, and identifying genes with the highest correlation. To determine whether two gene modules possess similar expression patterns, a threshold is typically set to sift through and merge gene modules that are deemed similar when exceeding this threshold.

### Preprocessing of data and selection of differentially expressed genes

Utilizing the “Limma” R package, with |log2Fold change(FC)| > 0.3 and p < 0.05 as the selection criteria, 592 DEGs were identified within the GSE33814 dataset. Heatmaps and volcano plots for DEGs were generated using the “pheatmap” (24) and “ggplot2” (25) packages.

### Establishment of Venn diagram

The Venn diagram were constructed using the Evenn website (http://www.ehbio.com/test/venn/).

### Conducting functional enrichment analysis

In this research, the “ClusterProfiler” R package (26) was employed for Gene Ontology (GO) enrichment analysis, encompassing Biological Process (BP), Molecular Function (MF), and Cellular Component (CC), as well as Kyoto Encyclopedia of Genes and Genomes (KEGG) and Disease Ontology (DO) functional enrichment analysis. A p-value of <0.05 was considered statistically significant.

### Application of machine learning for screening hub genes

LASSO regression is a regression analysis method that enhances the predictive accuracy of models by conducting variable selection and adjusting complexity through the compression of regression coefficients. A notable advantage of LASSO regression is its ability to simultaneously retain valuable features while compressing coefficients of irrelevant or less important features to zero, thus serving not only predictive purposes but also facilitating variable selection and model interpretation. Specifically, LASSO regression is achieved by introducing a regularization term, Lambda, to the foundation of Ordinary Least Squares (OLS) regression. The regularization term, constituting the sum of the absolute values of all regression coefficients, allows control over the magnitude of the regression coefficients. When the coefficient of the regularization term is adequately large, certain regression coefficients will be reduced to zero, thereby enabling feature selection.

SVM-RFE is a technique employed for feature selection, utilizing SVM to recursively eliminate the least important features. SVM-RFE operates through an iterative process, wherein the least crucial feature is removed at each step based on the coefficients of the SVM model, then an SVM model is rebuilt using the remaining features. This process persists until the desired number of features is attained. The technique offers the advantage of selecting a highly informative set of features within high-dimensional data, thus enhancing the model’s generalization capability.

RandomForest is an ensemble learning method that enhances predictive accuracy and robustness by aggregating the predictive results of multiple decision trees. The RandomForest algorithm can be applied to both classification and regression problems. The algorithm derives its name from its working principle: during the training process, the RandomForest randomly selects features from the feature set and constructs numerous decision trees. Each tree is trained on an independent subset of samples, obtained through bootstrap sampling. The predictive process of the RandomForest is as follows: in classification problems, a new input sample is predicted individually by all the decision trees, and the final prediction is determined by majority voting; in regression problems, the final prediction is the average of the predictions made by all the decision trees.

LASSO regression is executed using the “glmnet” package (27). SVM-RFE is realized utilizing the “e1071” (28) and “caret” packages (29). RandomForest is implemented using the “randomForest” package (30).

### Establishment of protein-protein interaction network

We utilized the “STRINGdb” package (31) to construct a PPI network and used the “igraph” package (32) to visualize the PPI of hub genes, based on betweenness values. Simultaneously, we used the GeneMANIA website (http://genemania.org/) to build a protein-protein interaction network.

### Analysis of immune infiltration

CIBERSORT is a computational biology tool that employs a deconvolution algorithm to estimate the proportions of 22 immune cell types in both NAFLD and control groups, based on gene expression data. It is capable of quantitatively estimating the presence of immune cells in tissue samples without direct measurement of immune cell infiltration.

### Construction of nomogram and analysis of ROC curve

A nomogram is a graphical tool widely used to predict the probability of a particular outcome based on a series of variables. In this study, the nomogram was constructed using the “rms” package (33).

The ROC curve is a graphical tool utilized to evaluate the predictive performance of hub genes. It illustrates the performance of hub genes across all possible classification thresholds by plotting the relationship between the True Positive Rate (TPR) and False Positive Rate (FPR) at various thresholds. In this research, the ROC curve was developed using the “pROC” package (34).

### Conducting gene set enrichment analysis of hub genes and single sample gene set enrichment analysis of hallmark gene sets

We conducted a single-gene GSEA to investigate the potential roles of hub genes. ssGSEA is employed to extract the enrichment score of specific gene sets from the gene expression data of a single sample. ssGSEA considers the rankings of all genes, not just those that are significantly differentially expressed. The ssGSEA scores can be interpreted as the rank of gene expression relative to background gene expression within a given gene set. The Hallmark gene sets, created by the Molecular Signatures Database (MSigDB) project at the Broad Institute, aim to condense and reorganize the broader C2 Canonical pathways gene sets. Encompassing 50 distinct sets, each represents a specific biological process. The design of Hallmark gene sets seeks to clarify the relationship between gene function and biological processes. Each Hallmark gene set captures a specific biological state or process by summarizing multiple similar gene sets and extracting their common variation through Principal Component Analysis (PCA). This approach benefits from reduced redundancy and noise, enhancing the biological significance of the gene set. Combining ssGSEA with Hallmark gene sets aids in understanding the activity levels of various biological processes and pathways within a single sample.

### Processing of single-cell sequencing data

The single-cell RNA sequencing (scRNA-seq) dataset GSE189600 was downloaded from the GEO database, comprising three NASH samples and three healthy samples serving as control (35). The analytical process unfolded as follows: Post-Quality Control (QC), the 10x scRNA-seq data was converted into Seurat objects, followed by a reduction in feature dimensions utilizing PCA and Uniform Manifold Approximation and Projection (UMAP) to identify distinct cellular subgroups. Subsequently, marker genes within different clusters were detected, and various cell types were annotated, followed by functional enrichment analysis. The “Linnorm” (36), “scater” (37), “Seurat” (38) and “SingleR” (39) packages were utilized throughout this process.

### TF-miRNA-mRNA regulatory network

The NetworkAnalyst website (https://www.networkanalyst.ca/NetworkAnalyst/) encompasses numerous databases to predict potential Transcription Factors (TFs) and microRNAs (miRNAs). In the present study, Transcription factor targets were derived from the JASPAR database, and Comprehensive experimentally validated miRNA-gene interaction data were collected from the miRTarBase v8.0 database.

### Attie lab diabetes database

The BTBR ob/ob mouse model is extensively used in the study of Type 2 Diabetes (T2D) and obesity in laboratory settings. This model combines the characteristics of the BTBR strain with mutations in the leptin gene (ob/ob), which are key factors in the onset of obesity and diabetic symptoms. The database allows for the querying of gene expression in six critical tissues, including the islets, liver, adipose tissue, hypothalamus, gastrocnemius muscle, and soleus muscle, based on variables such as genetic obesity status (lean *vs* ob/ob), mouse strain (B6 *vs* BTBR), and different age stages (4 weeks old *vs* 10 weeks old). This study employs the mlratio as a metric to assess changes in gene expression, where mlratio refers to the base-10 logarithm of the ratio of gene expression in an experimental sample (individual mice) relative to a specific strain reference pool (B6 strain or BTBR strain). The reference pool data is derived from 20 mice per strain, including lean and ob/ob mice at ages of 4 weeks and 10 weeks, with five mice from each age group. Our research focuses on the liver tissue of 10-week-old lean and ob/ob mice from both B6 and BTBR strains, with statistical analysis and graphical representation conducted using GraphPad Prism 9.

### NASH mouse model

In this study, we utilized female C57BL/6 mice, aged between 6 to 8 weeks, and subjected them to a high-fat, high-cholesterol (HFHC) diet while administering intraperitoneal injections of CCl4. This regimen was maintained for a total duration of 17 weeks to establish a NASH mouse model. The CCl4 injections were given once weekly at a dosage of 0.32 µg/g. The HFHC diet, acquired from Dyets Inc, under the product code D18061501, is characterized as a Modified Western Diet with 41% sucrose and 1.25% cholesterol. The caloric content of the diet was distributed as follows: 17% from protein, 43% from carbohydrates, and 40% from fats.

### RT-qPCR

Liver tissues from wild-type (wt) mice and NASH models were thoroughly homogenized, and RNA was extracted using the TRIzol method. Subsequent reverse transcription and PCR processes were conducted using Vazyme’s reverse transcription kit (catalog number R323) and PCR kit (catalog number Q341), respectively. The reverse transcription was performed on the GeneAmp PCR System 9700 from Applied Biosystems, while PCR amplification was carried out on the LightCycler 480 II system from Roche. All primers were purchased from Sangon Biotech. The primer sequences for RT-PCR are as follows: GAPDH: forward AGGTCGGTGTGAACGGATTTG, reverse TGTAGACCATGTAGTTGAGGTCA;CDKN1B: forward AGCAGTGTCCAGGGATGAGGAA, reverse TTCTTGGGCGTCTGCTCCACAG;TFAM: forward GAGCAGCTAACTCCAAGTCAG, reverse GAGCCGAATCATCCTTTGCCT. All experiments were performed in triplicate. Melting curve analysis confirmed the specificity of the PCR amplification as single peaks. The Ct values obtained were analyzed using the 2-ΔΔCt method, with GAPDH serving as the standard, to calculate the relative RNA expression levels.

### Western blot

Liver tissues from wild-type (wt) and NASH model mice were finely minced and then subjected to protein extraction via the RIPA method. The expression levels of β-actin and tubulin were normalized using their grayscale values measured by ImageJ. Polyacrylamide gels were prepared using the One-Step PAGE Gel Fast Preparation Kit (15%) from Vazyme (catalog number E305), with the 180 kDa Prestained Protein Marker from Vazyme (catalog number MP102) used for molecular weight estimation. Electrophoresis and membrane transfer were conducted using the PowerPac Basic Power Supply from BIO-RAD. Blocking was performed with 5% BSA. Primary antibodies were diluted as follows: β-actin at 1:1000 from Servicebio (catalog number GB15001-100), tubulin at 1:5000 from Affinity Biosciences (catalog number T0023), CDKN1B at 1:1000 from BIOSS (catalog number bs-0742R), and TFAM at 1:1000 from Proteintech (catalog number 22586-1-AP). Imaging was done using the Tanon 4800 system. Grayscale values for all bands were acquired with ImageJ, and the relative protein expression levels were determined using β-actin and tubulin as standards. Statistical analysis and graphical representation were performed using GraphPad Prism 9.

### Statistical analysis

R software (version 4.2.2; https://www.r-project.org/) and GraphPad Prism 9 were employed for all statistical analyses and graph generation. The Wilcoxon test and Student’s t-test were utilized to compare intergroup differences. ROC (Receiver Operating Characteristic) curves were used to evaluate the predictive performance of candidate genes used to construct predictive models. A P-value <0.05 was considered to indicate statistical significance.

## Results

### Implementation of WGCNA and identification of key module genes

**Figure 2:**
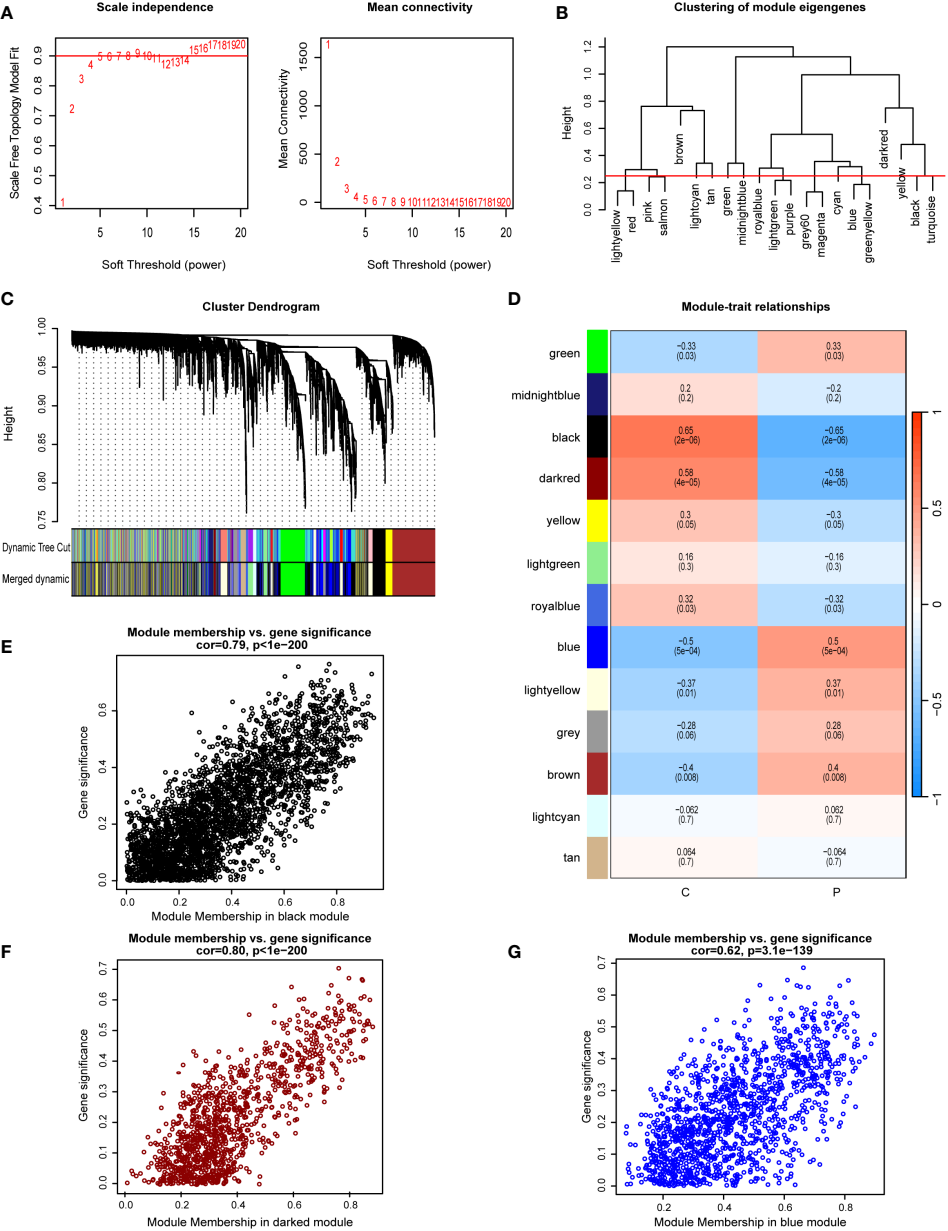
Implementation of WGCNA and identification of key module genes. **(A)** A soft-thresholding power (β) was set at 15, ensuring a scale-free R^2 ^= 0.9. **(B)** Hierarchical clustering dendrogram of module eigengenes. **(C)** The cluster dendrogram of co-expression network modules from WGCNA depending on a dissimilarity measure. **(D)** Module-trait relationships between: comparing the control group (C) with the NAFLD group (P). **(E)** The scatterplot for the black module displays the relationship between module membership and gene significance. **(F)** The scatterplot for the darkred module displays the relationship between module membership and gene significance. **(G)** The scatterplot for the blue module displays the relationship between module membership and gene significance.

WGCNA was used to identify modules most significantly correlated within the GSE33814 dataset. A soft-thresholding power (β) was set at 15, ensuring a scale-free R^2^ = 0.9, to accommodate gene expression relevant to a scale-free network (**Figure 2A**). The clustering of module eigengenes is employed to display the results of hierarchical clustering. In the diagram, ‘Height’ denotes the dissimilarity between clusters. When two clusters join at a lower height, it indicates greater similarity between them; conversely, a higher joining point suggests greater dissimilarity. Color labels represent different modules, each typically comprising a group of genes with similar expression patterns. This allows for the identification of gene modules with similar expression patterns (**Figure 2B**). The Cluster Dendrogram is also utilized to demonstrate the outcomes of hierarchical clustering analysis. The top of the dendrogram features a black line, with each bifurcation representing a split or merge in the clustering process. Colored bands denote different clusters obtained through the Dynamic Tree Cut method, with each color representing a cluster and the horizontal length indicating the number of objects within each cluster (**Figure 2C**). Module-trait relationships illustrate the associations between different gene modules (indicated by colors) and NAFLD, with each grid representing the correlation between a specific gene module and NAFLD (**Figure 2D**). A total of 13 gene co-expression modules were identified in the Module-trait relationships between the NAFLD group and the control group (**Figure 2D**). Notably, the black module (cor=-0.65, p=2e-6), darkred module (cor=-0.58, p=4e-05), and blue module (cor=0.5, p=5e-04) demonstrated the most significant correlations. The scatterplot for the black module displays the relationship between module membership and Gene Significance, with a correlation coefficient (cor) of 0.79 (p<1e-200). This indicates that as a gene’s membership in the black module increases—denoting higher similarity in expression patterns with other genes in the module—its association with NAFLD and its importance in the studied traits also increases (**Figure 2E**). Similar conclusions can be drawn from the scatterplots for the darkred and blue modules, which have correlation coefficients of 0.80 (p<1e-200) and 0.62 (p=3.1e-139), respectively (**Figures 2F, G**). Within these three modules, a total of 5361 genes were screened.

### Preprocessing of data and selection of DEGs

**Figure 3:**
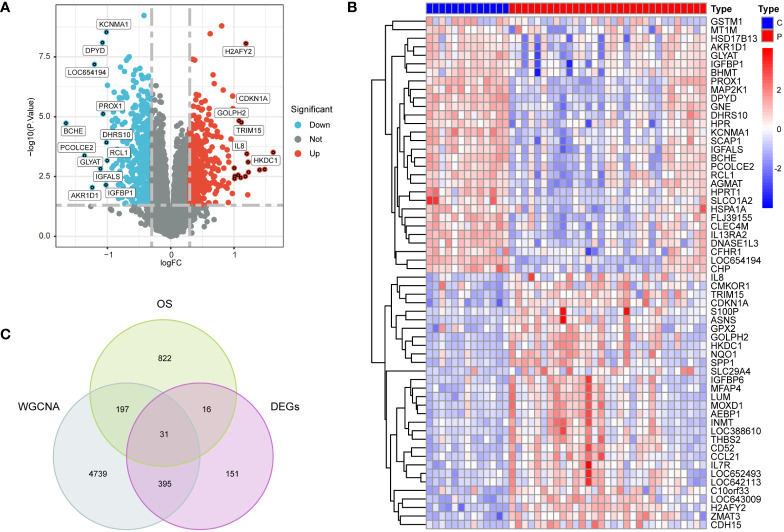
Preprocessing of data and selection of DEGs. **(A)** Volcano plots of DEGs in GSE3814, |log2 fold change (FC)| > 0.3 and p < 0.05 as selection criteria **(B)** heatmaps of DEGs in GSE33814: comparing the control group (C) with the NAFLD group (P). **(C)** 31 intersection genes of OS,WGCNA and DEGs.

Utilizing |log2 fold change (FC)| > 0.3 and p < 0.05 as selection criteria, 592 DEGs were identified within GSE33814. Volcano plots were crafted using the “ggplot2” R package (**Figure 3A**), the vertical lines represent |log2 fold change (FC)| > 0.3, and the horizontal line represents p < 0.05.Heatmaps were generated with the “pheatmap” R package (**Figure 3B**). Employing a Relevance score greater than 7 as a selection criterion in the Genecard database, 1065 genes related to oxidative stress were identified. The intersection of genes derived from the three methods yielded 31 intersection genes of NAFLD and OS (**Figure 3C**).

### Conducting functional enrichment analysis of 31 intersection genes of NAFLD and OS

**Figure 4:**
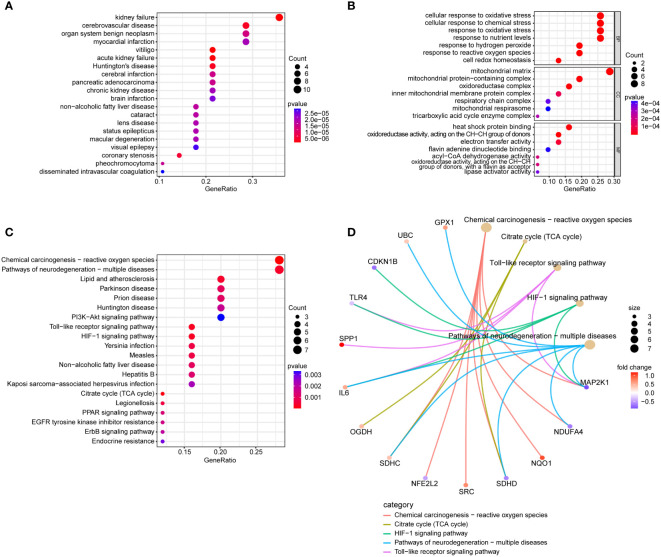
Conducting functional enrichment analysis of 31 intersection genes of NAFLD and OS. **(A)** The DO enrichment analysis reveals the diseases most significantly associated with the 31 intersecting genes. **(B)** The GO enrichment analysis elucidates the functional roles of the 31 intersecting genes from three perspectives: BP, CC, and MF. **(C)** The KEGG enrichment analysis bubble plot displays the signaling pathways most closely related to the 31 intersecting genes. **(D)** The KEGG enrichment analysis circular network plot presents a network of relationships between some genes and their associated signaling pathways.

In the DO enrichment analysis, kidney failure and cerebrovascular disease were significantly enriched (**Figure 4A**). In the GO enrichment analysis (**Figure 4B**), BP categories were enriched in cellular response to oxidative stress, cellular response to chemical stress, response to oxidative stress, and response to nutrient levels. CC categories were enriched in mitochondrial matrix and mitochondrial protein-containing complex, and MF categories were enriched in heat shock protein binding, oxidoreductase activity, acting on the CH-CH group of donors, and electron transfer activity. In the KEGG functional enrichment analysis (**Figure 4C**), Chemical Carcinogenesis - Reactive Oxygen Species, Pathways of Neurodegeneration - Multiple Diseases, HIF-1 Signaling Pathway, and Toll-like Receptor Signaling Pathway were significantly enriched and the genes enriched in these pathways are illustrated (**Figures 4C, D**).

### Application of machine learning for screening hub genes

**Figure 5:**
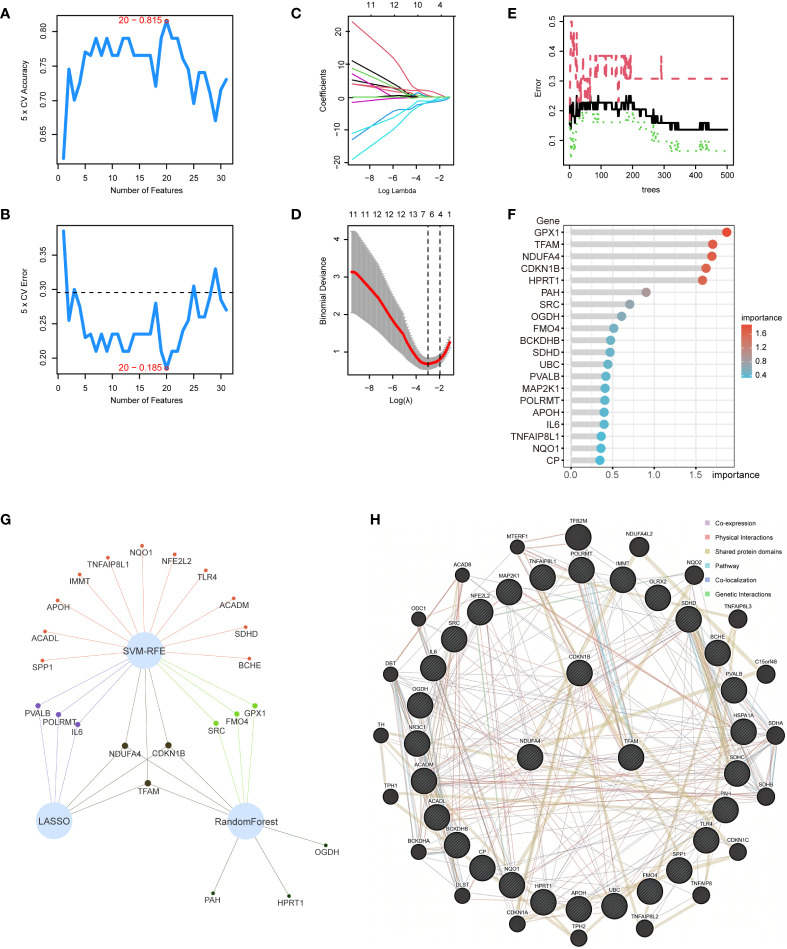
Application of machine learning for screening hub genes. **(A, B)** SVM-RFE algorithm to extract 20 genes. **(C, D)** 6 genes were identified through the LASSO regression algorithm. **(E, F)** RandomForest algorithm selected 9 genes. **(G)** Venn network to intersect 3 gene subsets. **(H)** 3 hub genes and other intersection genes of NAFLD and OS were explored within the PPI network.

Within the 31 intersection genes of NAFLD and OS, we first utilized the SVM-RFE algorithm to extract 20 genes (**Figures 5A, B**). Subsequently, 6 genes were identified through the LASSO regression algorithm (**Figures 5C, D**). Following this, the RandomForest algorithm selected 9 genes (**Figures 5E, F**). Ultimately, by employing a Venn network to intersect these gene subsets, we identified 3 genes: CDKN1B, NDUFA4, and TFAM (**Figure 5G**). Simultaneously, the interaction relationships between these 3 hub genes and other intersection genes of NAFLD and OS were explored within the PPI network (**Figure 5H**).

### GSEA of hub genes and ssGSEA of hallmark gene sets

The Gene Set Enrichment Analysis (GSEA) plots provide insights into the biological processes enriched during high and low expressions of individual genes. This enrichment allows us to rank these processes and identify those with the significant differences. Such analyses are instrumental in revealing the molecular mechanisms underlying changes in biological states and the affected biological pathways. In these plots, the horizontal axis represents gene ranking within an ordered dataset, typically based on expression levels from high to low. The vertical axis shows the running enrichment score (ES) for the gene set. The ranked list metric at the bottom indicates the value used for gene ranking, which could be the signal-to-noise ratio, fold change, or other statistical measures of differential expression. The lines in the plots trace the path of the enrichment score across the ranked gene list for each gene set, while the vertical lines below the plot signify the positions of genes from the gene set within the ranked list.

**Figure 6:**
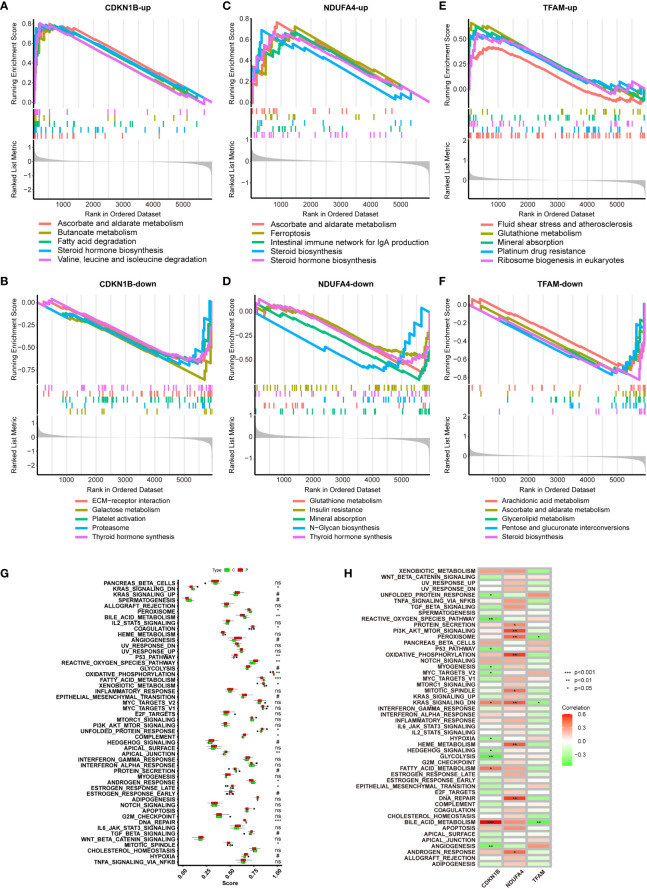
GSEA of Hub Genes and ssGSEA of Hallmark Gene Sets. **(A, B)** GSEA analysis of CDKN1B-up and CDKN1B-down. **(C, D)** GSEA analysis of NDUFA4-up and NDUFA4-down. **(E, F)** GSEA analysis of TFAM-up and TFAM-down. **(G)** ssGSEA Hallmark Gene Sets functional enrichment analysis results for the NAFLD group and the control group. **(H)** ssGSEA Hallmark Gene Sets functional enrichment analysis of the three hub genes.

**Figure 6A** demonstrates gene sets associated with upregulated genes linked to CDKN1B. The top of the ranked list features enriched gene sets including ascorbate and aldarate metabolism, butanoate metabolism, fatty acid degradation, steroid hormone biosynthesis, and the degradation of valine, leucine, and isoleucine. **Figure 6B** presents gene sets associated with downregulated genes linked to CDKN1B, including ECM-receptor interaction, galactose metabolism, platelet activation, proteasome, and thyroid hormone synthesis. **Figure 6C** illustrates gene sets related to genes upregulated in connection with NDUFA4, encompassing ascorbate and aldarate metabolism, ferroptosis, the intestinal immune network for IgA production, and steroid biosynthesis. **Figure 6D** reveals gene sets corresponding to genes downregulated with NDUFA4, highlighting glutathione metabolism, insulin resistance, mineral absorption, N-glycan biosynthesis, and thyroid hormone synthesis. **Figure 6E** shows gene sets related to upregulated genes in association with TFAM, with the top-ranked list showing enrichment in gene sets such as fluid shear stress and atherosclerosis, glutathione metabolism, mineral absorption, platinum drug resistance, and ribosome biogenesis in eukaryotes. **Figure 6F** displays gene sets linked to downregulated genes in connection with TFAM, featuring gene sets like arachidonic acid metabolism, ascorbate and aldarate metabolism, glycerolipid.

ssGSEA, is a method designed to calculate the degree of enrichment between gene expression data of a single sample and a predefined set of genes. In contrast to traditional GSEA, which compares groups of samples or conditions, ssGSEA allows for scoring each individual sample independently. This proves particularly valuable in revealing changes in biological processes within individual samples that do not show significant changes at the group mean level, especially useful in samples with substantial heterogeneity. It does not necessitate a control group and is applicable to a variety of gene expression data types, including those from public databases. The operational procedure is as follows: firstly, it ranks all genes based on their expression levels; then, for each gene set, ssGSEA calculates an enrichment score that reflects the relative positioning and distribution of genes within that set in the ranking. This score is derived by accumulating the scores of genes within the gene set while subtracting the scores of genes not included in the set; ultimately, this score may be normalized to allow comparisons across different samples or gene sets. A Hallmark Gene Set denotes a group of genes whose patterns of expression have specific biological significance, such as being indicative of certain cell types, diseases, or biological processes, and are often identified through the analysis of experimental data. In summary, a Hallmark Gene Set provides a predefined list of genes that are considered biologically relevant; ssGSEA is an analytical tool that uses these sets to quantitatively assess the expression of these gene sets in individual samples. By doing so, ssGSEA can reveal the unique biological characteristics inherent to each sample. By employing this method, we can finally determine the significant differences in biological processes between the control group and the NAFLD group (**Figure 6G**), and ascertain the specific biological processes in which the three hub genes differ significantly (**Figure 6H**).

### Clinical studies of the hub genes

**Figure 7:**
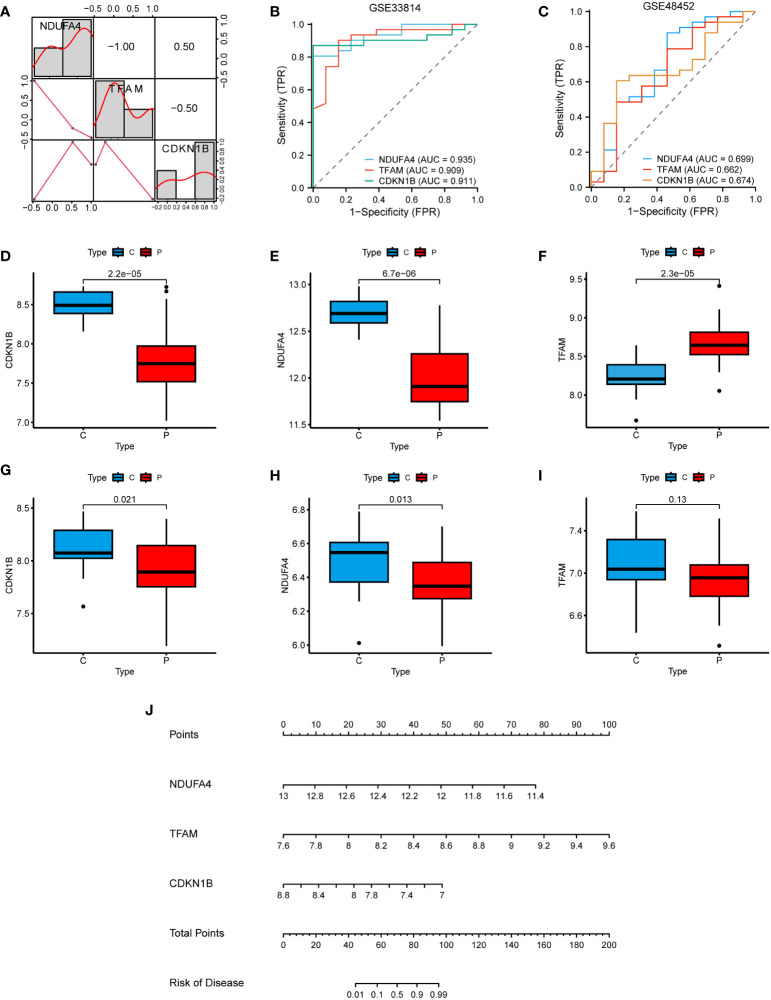
Clinical studies of the hub genes. **(A)** Correlation heatmap of the 3 hub genes. **(B)** ROC curve in the GSE33814. **(C)** ROC curve in the GSE48452. **(D–F)** Hub genes expression in the GSE33814: comparing the control group (C) with the NAFLD group (P). **(G–I)** Hub genes expression in the GSE48452: comparing the control group (C) with the NAFLD group (P). **(J)** Nomogram for the diagnosis of NAFLD based on the hub genes.

In the correlation heatmap, we observed a positive correlation between CDKN1B and NDUFA4, while TFAM was negatively correlated with them (**Figure 7A**). In the GSE33814 dataset, the diagnostic value of these three hub genes was further validated through the ROC curve. Specifically, NDUFA4 (AUC: 0.935), TFAM (AUC: 0.909), and CDKN1B (AUC: 0.911) demonstrated significant diagnostic value for NAFLD (**Figure 7B**). Similar results were obtained in the GSE48452 validation dataset (**Figure 7C**). Through the investigation of the GSE33814 dataset, we discovered that CDKN1B and NDUFA4 expressions were reduced in NAFLD, whereas TFAM expression was elevated (**Figures 7D–F**). These findings were validated in the GSE48452 dataset (**Figures 7G–I**). Additionally, we constructed a nomogram to predict the incidence of NAFLD (**Figure 7J**). These results suggest that the three hub genes present a satisfactory performance in diagnosing NAFLD.

### Analysis on immunization: immune infiltration analysis and processing of single-cell sequencing data

**Figure 8:**
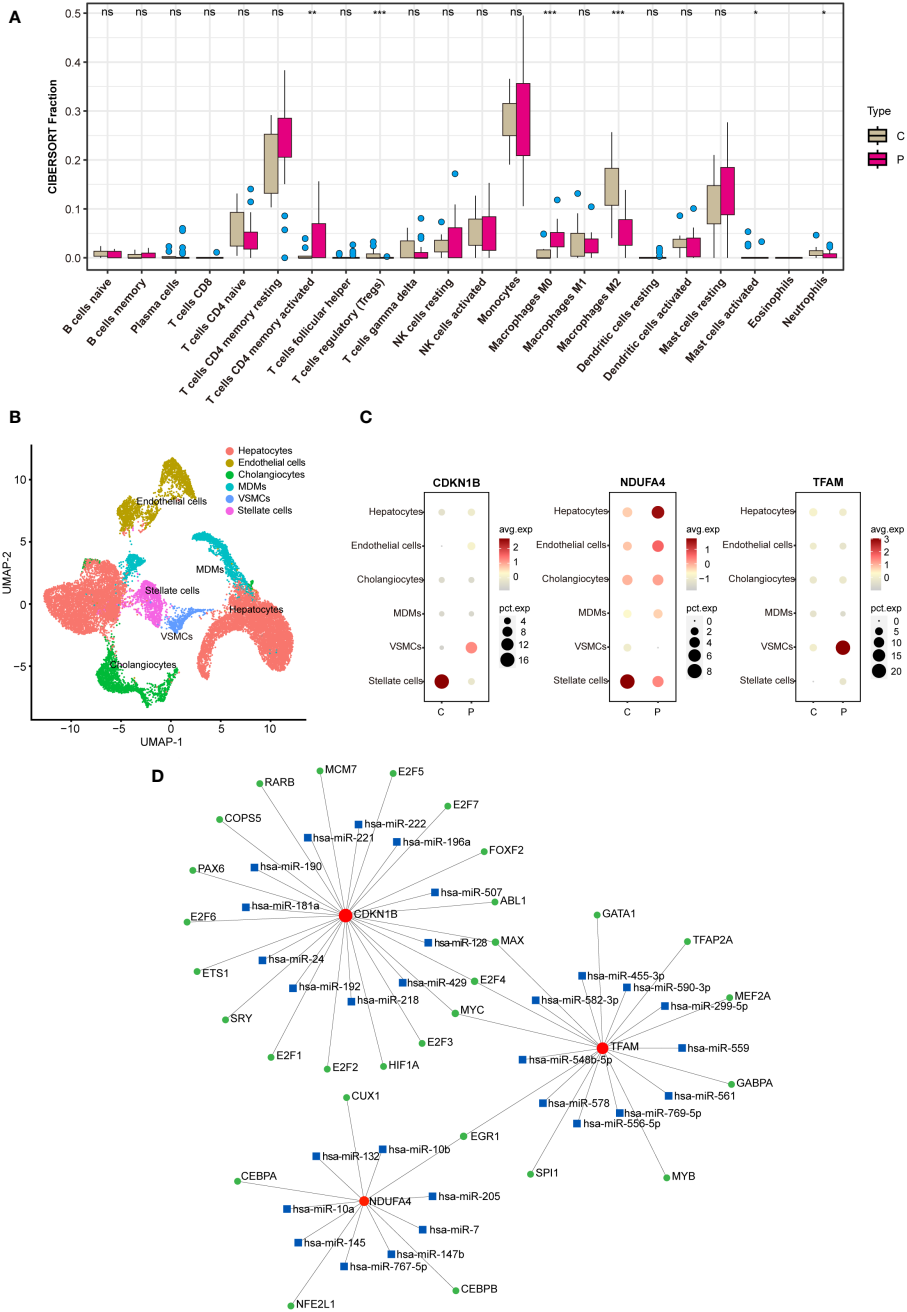
Analysis on immunization and TF-miRNA-mRNA regulatory network. **(A)** Immune infiltration analysis between the NAFLD group and the control group. **(B)** Analysis of the single-cell RNA sequencing dataset GSE189600. **(C)** Hub genes enrichment in immune cells. **(D)** TF-miRNA-mRNA regulatory network. In B, C, and D, “C” represents the control group, and “P” represents the NAFLD group. "ns" indicates not significant, "*" indicates P<0.05, "**" indicates P<0.01, "***" indicates P<0.001.

Utilizing the CIBERSORT algorithm for immune infiltration analysis, significant differences were observed between the control and NAFLD groups in Tregs, M0 macrophages, M2 macrophages, T cells CD4 memory activated, activated mast cells, and neutrophils (**Figure 8A**). Analysis of the single-cell RNA sequencing dataset GSE189600 determined the distribution of three hub genes across six cell clusters (**Figure 8B**). Significant disparities were identified between the control and NAFLD groups for CDKN1B in stellate cells and vascular smooth muscle cells (VSMCs). For NDUFA4, notable differences were observed between the control and NAFLD groups in stellate cells and hepatocytes. In the case of TFAM, the control and NAFLD groups demonstrated significant variation in VSMCs (**Figure 8C**).

### TF-miRNA-mRNA regulatory network

Utilizing the JASPAR database, potential transcription factors were predicted on the NetworkAnalyst website, while possible miRNAs were foreseen using the miRTarBase v8.0 database. Subsequently, a regulatory network map was constructed based on their interactive relationships (**Figure 8D**). Transcription factors (TFs) are proteins that typically bind to specific DNA sequences to control the transcription of genetic information from DNA to mRNA, represented by green circles in the network. MicroRNAs (miRNAs) are short non-coding RNA molecules that bind to complementary sequences on target mRNAs, regulating gene expression post-transcriptionally, often resulting in mRNA degradation or repression of translation, depicted by blue squares. Messenger RNAs (mRNAs) are the final transcripts that carry genetic information from DNA—transcribed by the action of TFs—to the ribosome, where proteins are synthesized. Lines within the network indicate interactions or regulatory influences between these entities, with the direction of regulation (from TF to mRNA or from miRNA to mRNA) typically denoted by lines originating from the regulator and pointing towards the target. These networks are crucial for understanding the complex layers of gene regulation within cells, elucidating how genes are switched on or off, how miRNA fine-tunes this regulation, and the intricate balance that maintains normal cellular function or contributes to disease when dysregulated.

### Animal experimentation

**Figure 9:**
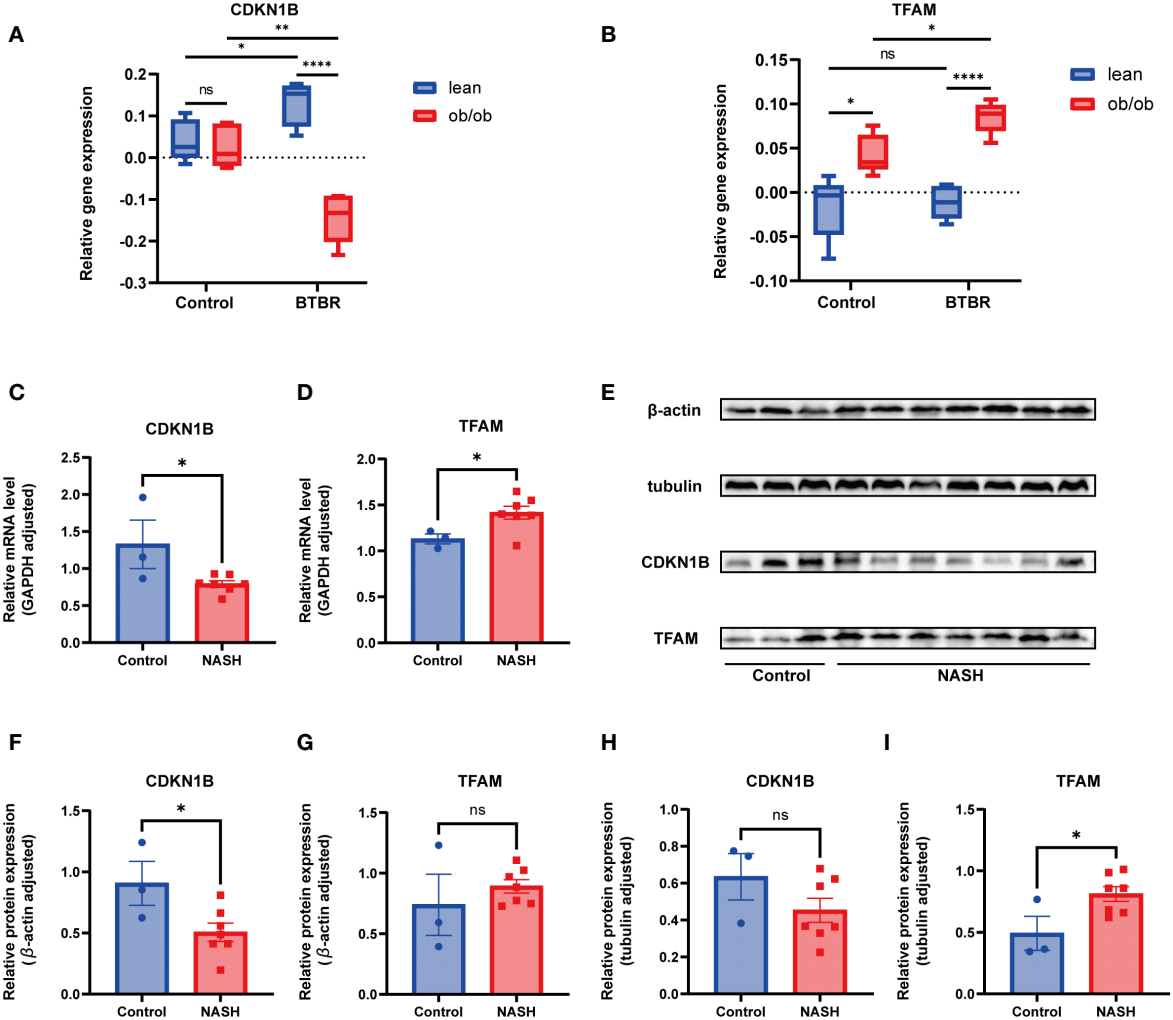
Animal experimentation **(A)** Comparison of CDKN1B relative gene expression in lean and ob/ob groups of 10-week-old C57BL/6 and BTBR strain mice, using mlratio to quantify changes. **(B)** Comparison of TFAM relative gene expression in lean and ob/ob groups of 10-week-old C57BL/6 and BTBR strain mice, using mlratio to quantify changes. **(C)** Relative mRNA levels of CDKN1B in control and NASH mice. **(D)** Relative mRNA levels of TFAM in control and NASH mice. **(E)** Comparison of protein expression levels for CDKN1B and TFAM. **(F)** Statistical analysis of CDKN1B protein expression, standardized by β-actin. **(G)** Statistical analysis of TFAM protein expression, standardized by β-actin. **(H)** Statistical analysis of CDKN1B protein expression, standardized by tubulin. **(I)** Statistical analysis of TFAM protein expression, standardized by tubulin. "ns" indicates not significant, "*" indicates P<0.05, "**" indicates P<0.01, "***" indicates P<0.001.

Considering the prevalent obesity and diabetes symptoms in NAFLD patients, this study utilized the Attie Lab Diabetes Database BTBR ob/ob mouse model to select liver tissues from 10-week-old C57BL/6 (control) and BTBR mice, categorizing them into lean and ob/ob groups, to investigate the expression of hub genes under various conditions. The findings indicated significant differences in the expression of CDKN1B and TFAM genes between the control and BTBR strains, as well as between the lean and ob/ob mice, aligning with our expectations (**Figures 9A, B**).

Subsequently, this research focused on a NASH mouse model, representing a more advanced stage of NAFLD, employing RT-qPCR and Western Blot techniques to examine the expression of these key genes at RNA and protein levels, respectively. The RT-qPCR results revealed significant differences in the expression of CDKN1B and TFAM between the control and NASH groups, consistent with prior expression trend predictions (**Figures 9C, D**). At the protein level, the findings from the WB analysis corroborated those from RT-qPCR (**Figure 9E**), with subsequent statistical analysis conducted (**Figures 9F–I**).

## Discussion

NAFLD, a disease syndrome that encompasses NAFL and NASH, impacts nearly a quarter of the global population, with its prevalence escalating annually. Alarmingly, NASH possesses the potential to further progress into cirrhosis and hepatocellular carcinoma, triggering a cascade of complications and ultimately, may prove fatal, thereby imposing a substantial disease burden on society. In light of this, an in-depth understanding of NAFLD’s pathogenic mechanisms, formulation of appropriate therapeutic strategies, and identification of reliable diagnostic markers become paramount.

miRNAs are genes encoding small RNAs, predominantly functioning by inhibiting the translation of target mRNAs or inducing their degradation, thereby playing pivotal roles in the proliferation, development, and differentiation across numerous cell types, and is also involved in the progression of various diseases. The microRNA regulatory studies have been exhibited in the TF-miRNA-mRNA regulatory network we constructed (**Figure 8C**) and have been validated in numerous previously published papers.

Hepatotoxicity mediated by free radicals and demonstrated the therapeutic effect of antioxidants against free radical-mediated NAFLD. Moreover, epidemiological statistics on the liver have confirmed that changes in the redox state of NAFLD are closely related to an increased subsequent metabolic risk. According to the “second hit” and “multiple hit” theories, oxidative stress appears to be one of the most crucial mechanisms leading to liver injury in NAFLD.

The CDKN1B gene encodes the p27 protein, which plays a crucial role in regulating cell growth, differentiation, cytoskeletal dynamics, and cell division. A reduction in p27 protein is associated with the invasiveness of various human tumors, such as colon cancer, breast cancer, prostate cancer, and ovarian cancer (40). Hepatic fibrosis and hepatocellular carcinoma are closely related to CDKN1B. The accumulation of the Extracellular Matrix (ECM) in the liver leads to the onset of liver fibrosis. Excessive production of ECM by activated hepatic stellate cells and myofibroblasts is considered the primary mechanism inducing liver fibrosis, which may further develop into cirrhosis and hepatocellular carcinoma. miR-221/222 is considered a new indicator of stellate cell activation and liver fibrosis progression. The expression of miR-221/222 is positively correlated with the progression of liver fibrosis and significantly associated with the expression of Col1A1 and αSMA mRNA. The expression of miR-221/222 has been validated in human fibrotic liver samples and mouse models of liver fibrosis. They interact with CDKN1B and inhibit the expression of CDKN1B mRNA and protein in human stellate cell line LX-2. The expression of miR-222 in stellate cells may be regulated by NF-κB activation (41). The overexpression of miR-221/222 promotes cancer cell proliferation, most likely through their regulation of the CDKN1B expression (42). The upregulation of miR-221/222 can promote the growth of hepatocellular carcinoma (HCC) cells by increasing the number of S-phase cells, and the oncogenic activity of miR-221 is believed to be realized through the regulation of CDKN1B (42, 43). CDKN1B has been validated as a target of miR-221, and the CDKN1B gene is directly associated with HCC proliferation (44). F. Fornari et al. (45) observed that CDKN1B gene expression was downregulated in 77% of HCC samples, and the downregulation of CDKN1B affected the prognosis of HCC. In human HCC, the downregulation of CDKN1B showed prognostic significance associated with advanced tumor stages, lower survival rates, and HCC recurrence (46). HCC represents the terminal stage of NAFLD, suggesting that the regulatory mechanism of miR-221/222 on CDKN1B may play a vital role in the etiology of NAFLD. These findings provide a basis for developing potential therapeutic strategies for liver fibrosis and liver cancer.

NDUFA4 has been relatively underexplored. Initially, NDUFA4 was identified as a component of the mitochondrial respiratory complex I. However, subsequent studies revealed that NDUFA4 is actually associated with complex IV rather than complex I (47). This gene demonstrates significant tissue-specific expression in the liver and brain (48). NDUFA4 is a target of miR-147, and the inhibition of miR-147, coupled with the overexpression of NDUFA4, can induce mitochondrial damage and renal tubular cell death (49). A deficiency in NDUFA4 expression can exacerbate oxidative stress, further predisposing to the onset of diabetes (50). MiR-210 promotes the pathogenesis of obesity-induced diabetes in mice by targeting NDUFA4 gene expression (51). MiR-210-3p accelerates cardiomyocyte apoptosis and impairs mitochondrial function by targeting NDUFA4, contributing to the cardiac dysfunction induced by sepsis (52). In the liver, NDUFA4 may also play a role in disease onset through mechanisms related to mitochondrial dysfunction.

TFAM as a pivotal structural protein of mammalian nuclei, serves as a transcription activator, specifically stimulating certain mitochondrial transcription initiation points (53). This protein is integral to various processes, including the transcription and replication of mitochondrial DNA (mtDNA), its packaging into nucleoid structures, and playing an indispensable role in the regulation of mtDNA copy numbers. Notably, an overexpression of TFAM, exceeding normal physiological levels, can directly lead to postnatal death and mitochondrial functional impediments. Experimental evidence reveals that mice with high TFAM expression typically exhibit smaller sizes and weaker physical conditions compared to their wild-type littermates, with significantly reduced liver, heart, and kidney volumes. Further research has also disclosed an increase in lipid accumulation in the liver tissues of mice with TFAM overexpression, potentially attributable to the dysregulation of lipid metabolism induced by the upregulation of mitochondrial protease interference pathways (54). Variations in TFAM expression have also been observed in studies of other liver diseases. For instance, in a study related to alcoholic liver disease, the hepatic TFAM levels in mice fed with ethanol rose by 30% compared to the control group fed with water (55). Meanwhile, studies of human normal and malignant liver tissues and cell lines demonstrate that TFAM expression trends upward in Hepatocellular Carcinoma cells resistant to drugs. However, TFAM is only upregulated in a small portion of HCC patients, and inhibiting TFAM can suppress the growth and survival of HCC cells, thereby enhancing the effectiveness of chemotherapy (56).

While the importance of TFAM in maintaining mtDNA and facilitating mitochondrial biogenesis is widely acknowledged, the interactions between TFAM and certain miRNAs in the context of diseases remain shrouded in mystery. For example, a deficiency in human TFAM has been identified as a catalyst for mitochondrial dysfunction and a reduction in nucleoid formation, culminating in fatal liver failure (57). After TFAM depletion, its roles, both as an oncogene and a tumor suppressor, have been observed (58, 59). TFAM is identified as a direct target of miRNA-590-3p; in bladder cancer, a downregulation of miRNA-590-3p expression correlates with a marked increase in TFAM expression (60), while in colon cancer, an elevation in miRNA-590-3p expression is associated with a significant decrement in TFAM expression (61). Furthermore, factors such as sex, age, and diet can influence TFAM expression. For instance, TFAM protein levels in the livers of female rats are quadruple those in males, a sexual dimorphism fundamentally attributed to the females’ heightened degree of mitochondrial differentiation, which leads to superior substrate oxidation capability and efficiency (62). It is noteworthy that TFAM protein expression diminishes progressively with age, a process that can be fully mitigated through calorie restriction (CR) (63). In conclusion, the exact mechanisms by which TFAM functions in disease onset remain intricate and necessitate further exploration.

Transcription factors such as SPI1, ETS1, and CEBPA have been identified as promising targets for the prevention and treatment of NASH (64). These transcription factors are integral components of a complex regulatory network involving TF-miRNA-mRNA interactions, highlighting the sophisticated molecular interplay underlying NASH pathogenesis. CEBPA is linked to the regulation of NDUFA4, a component of the mitochondrial respiratory chain, suggesting a role in metabolic efficiency and oxidative stress response. SPI1’s regulation of TFAM, a key factor in mitochondrial DNA maintenance and transcription, points to its importance in mitochondrial biogenesis and function. ETS1’s influence on CDKN1B implicates it in cell cycle regulation and potentially in the control of hepatocyte proliferation and apoptosis, processes central to NASH progression and liver regeneration.

In our study, we employed immunoinfiltration analysis techniques to investigate the disparities in the immune cell composition between patients with NAFLD and healthy control groups. Significant differences were observed across several immune cell subpopulations, including neutrophils, macrophages, regulatory T cells (Tregs), and mast cells. Further, single-cell sequencing technology revealed expression pattern discrepancies in three hub genes within specific cellular subpopulations, such as hepatic stellate cells and vascular smooth muscle cells (VSMCs), suggesting their potential key regulatory roles in hepatic pathological processes. Notably, these cells play a decisive role in the development of inflammatory damage, hepatocyte injury, and liver fibrosis induced by oxidative stress.

Moreover, our comprehensive bioinformatics enrichment analyses identified multiple signaling pathways closely associated with the pathogenesis of NAFLD, related to oxidative stress. We also uncovered a series of critical biological processes, including dysregulated lipid metabolism, imbalance in inflammatory response regulation, and extracellular matrix remodeling. The aberrant regulation of these pathways and biological processes offers new insights into the pathophysiological foundation of NAFLD.

Nevertheless, the present study has not yet conducted in-depth mechanistic validations of these findings. Future research should explore the causal relationships between these central genes and the characteristics of immune cell infiltration, as well as their specific roles in the progression of NAFLD, through *in vivo* and *in vitro* experimental models. Additionally, the current study lacks direct experimental evidence at the cellular level, necessitating further validation of these genes’ roles and importance in the progression of NAFLD through functional experiments, such as gene knock-out, overexpression studies, and immunohistochemical staining. Through these extensive experimental investigations, we will be able to elucidate the pathological role of oxidative stress in non-alcoholic fatty liver disease more accurately and potentially develop new therapeutic targets.

## Data availability statement

The datasets presented in this study can be found in online repositories. The names of the repository/repositories and accession number(s) can be found in the article/**Supplementary Material**.

## Author contributions

HW: Conceptualization, Data curation, Formal analysis, Investigation, Software, Writing – original draft, Writing – review & editing. PH: Data curation, Formal analysis, Investigation, Software, Writing – original draft. WC: Data curation, Investigation, Writing – review & editing. TL: Data curation, Investigation, Methodology, Writing – review & editing. CH: Methodology, Writing – review & editing. YC: Methodology, Writing – review & editing. YZ: Methodology, Writing – review & editing. JW: Methodology, Writing – review & editing. QY: Conceptualization, Funding acquisition, Writing – original draft, Writing – review & editing. TZ: Conceptualization, Funding acquisition, Software, Writing – review & editing.
